# A vegetation configuration pattern with a high-efficiency purification ability for TN, TP, AN, AP, and COD based on comprehensive assessment results

**DOI:** 10.1038/s41598-018-38097-y

**Published:** 2019-02-20

**Authors:** Guirong Hou, Huaxing Bi, Xinxiao Yu, Guodong Jia, Dandan Wang, Zhenyao Zhang, Ziqiang Liu

**Affiliations:** 1 College of Soil and Water Conservation, Beijing Forestry University, 100083 Beijing, China; 2 Ji county station, Chinese National Ecosystem Research Network (CNERN), 100083 Beijing, China; 3 Beijing Collaborative Innovation Center for Eco-environmental Improvement with Forestry and Fruit Trees, 102206 Beijing, China; 4 Key Laboratory of State Forestry Administration on Soil and Water Conservation (Beijing Forestry University), 100083 Beijing, China; 5 Beijing Engineering Research Center of Soil and Water Conservation, (Beijing Forestry University), Beijing, 100083 China

## Abstract

To identify a vegetation configuration pattern with a high-efficiency purification ability for total nitrogen (TN), available nitrogen (AN), total phosphorous (TP), available phosphorous (AP), and chemical oxygen demand (COD) based on comprehensive assessment results, a water discharge experiment was performed in the Luan River in China with the following riparian forests: I, pure broad-leaved; II, mixed broad-leaved; III, mixed coniferous and broad-leaved; IV, mixed coniferous; and V, pure coniferous. From the riparian buffer zone to the river channel, the evaluation showed that pattern I had the highest purification ability at 1 m and 2 m; at a width of 4 m, pattern III had the highest purification ability; at a distance of 7 m, pattern V showed the highest purification ability; at 10 m, pattern IV showed the highest purification ability, pattern II the lowest. It is advisable to give priority to plant coniferous species from 0 m to 4 m from the river bank, while it is advisable to give priority to plant broad-leaved species from 4 m to 10 m from the river bank. We therefore recommend these vegetation configuration patterns in the development and management of runoff purification systems.

## Introduction

As water source pollution is a global challenge, improving the quality of surface runoff water is a focus of ecological restoration^1,2^. The accumulation of harmful substances from urban areas and overuse of fertilizer in agricultural are primary factors causing surface water quality degradation and water source pollution. Such degradation is not only a concern for agriculture and industry but also seriously affects the quality of life of residents^3,4^. Thus, identifying a vegetation configuration pattern with highly efficient purification ability for surface runoff water nutrients to prevent eutrophication and decrease risks for human health and aquatic ecosystems is imperative.

Planting buffer riparian vegetation is an effective strategy to intercept non-point-source pollution and improve river water quality. This measure plays an important role in reducing sediment and nutrient deposition in the river ecosystem protection zone, and it can effectively control the river pollution load^2,5,6^. A riparian vegetation buffer zone is a complex soil-plant-microorganism ecosystem that performs an ecological function by incorporating the synergistic effects of the physical, chemical and biochemical responses of natural ecosystems^7^. A vegetation buffer zone along a river bank can reduce surface water and groundwater pollution by filtering, absorbing, retaining, and depositing pollutants in addition to having physical, chemical and biological effects^8–10^. Previous studies have shown that a riparian zone vegetation buffer can reduce nitrogen (N) and phosphorus (P) concentrations in both surface runoff and subterranean water^11^, but there is a lack of comparative studies on the effect and control of nitrogen (total nitrogen (TN) and available nitrogen (AN)) and phosphorus (total phosphorous (TP) and available phosphorus (AP)) pollutants in surface runoff.

Riparian ecosystems are not only main pathway of transition of pollutants but also hubs of communication between aquatic and terrestrial ecosystems^11–13^, and they have an important function of improving water quality, mainly through their role in overland flow purification. The ecological service value of riparian vegetation buffer zones has been underestimated, and improving river water quality is an enormous challenge in China due to improper land use and agricultural pollution^14,15^.

Forests differ in their ability to purify water, and the chemistry of ground water changes significantly as it passes through a riparian vegetation buffer zone. Previous studies have shown that N and P concentration can be decreased by such buffer zones (Table 1). For example, Sharpley reported that the removal effectiveness of P from surface water is as high as 80% in a riparian zone dominated by pure forest^16^, and Schoonover suggested that the removal effectiveness of N from surface water can reach 78% and that for P is as high as 97% in riparian zones dominated by forest and herbaceous cover^17^. However, Guo reported that the removal effectiveness of N and P from surface water is 30% and 52%, respectively, in a riparian zone dominated by herbaceous cover^15^. Therefore, riparian vegetation buffer zones are critical for decreasing N (TN and AN) and P (TP and AP) levels^18,19^. Chemical parameters are measured to evaluate water quality and contamination with pollutants, aiding in the development of effective management strategies^4^.

**Table 1:** The removal effectiveness of nitrogen and phosphorus in riparian zones for surface water (SW) and ground water (GW).

Vegetation type	N		P		Reference
	Removal effectiveness (%)		Removal effectiveness (%)		
	SW	GW	SW	GW	
Forest			80		Sharpley^45^
Herbaceous	57				Dillaha *et al*.^46^
wetland		76			Clausen *et al*.^20^
Forest	98	39			Sabater *et al*.^47^
Herbaceous	60	98			Vidon and Hill^48^
Forest/Herbaceous	97		78		Schoonover *et al*.^17^
Wetland	60				Fox *et al*.^49^
Forest			30	15	Zaimes *et al*.^18^
Forest	77	98			Woodward *et al*.^50^
Forest	92.1		91.8		Mankin *et al*.^51^
wetland	34.9	43.81	62.05	74.81	Wang *et al*.^27^
Forest	83				Johnson *et al*.^52^
Forest/Herbaceous	98				Hill *et al*.^53^
Herbaceous	52		30		Guo *et al*.^15^
Forest/Herbaceous	42.9				Lin *et al*.^54^
Forest/Herbaceous	67		69		Liolios *et al*.^55^

The Luan River is an important watershed in northern China, a region of agricultural activities, with different riparian forest types distributed along the river. Approximately 3.5 million people live in Chengde City, and the Luan River is the main water source for the city. In addition, at the outlet of the Luan River is the Miyun Reservoir, which provides domestic water for Beijing City, where approximately 21.73 million people live. Therefore, preventing water source pollution has become a problem demanding prompt solution. A water discharge experiment was carried out to evaluate the purification effects of riparian vegetation patterns on the overland runoff nutrients in the Luan River in China.

Accordingly, the first objective of this research was to analyse the distribution characteristics of TN, TP, AN, AP, and chemical oxygen demand (COD) in five riparian vegetation configuration patterns in a riparian transect in the Luan River in China. The second objective was to identify a vegetation configuration pattern with a high-efficiency purification ability for TN, TP, AN, AP, and COD based on comprehensive assessment results.

## Materials and Methods

### Study area

**Figure 1:**
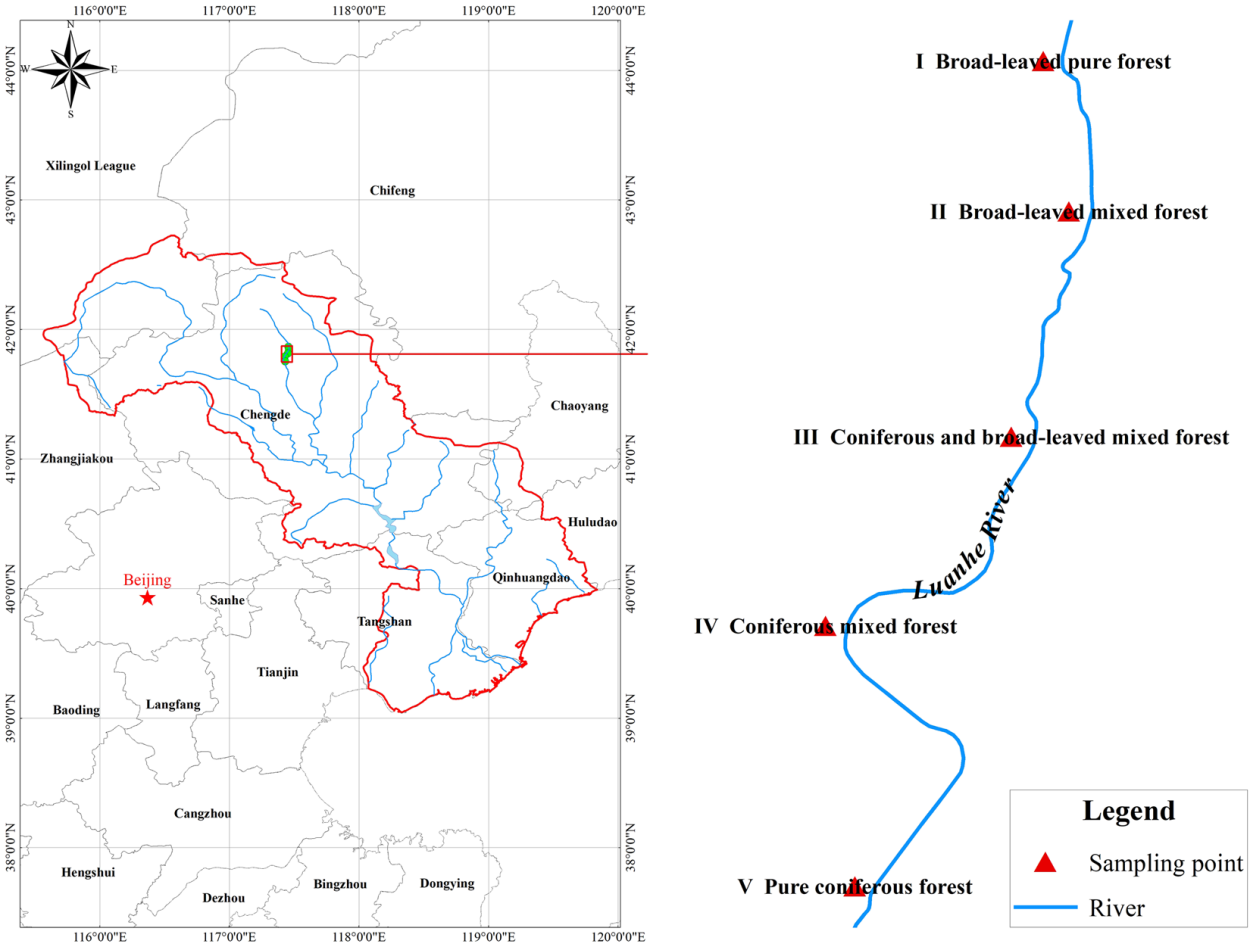
Location of Luan River study area. The figure of location of Luan River study area were generated with ArcGIS 10.1 (Environmental Systems Research Institute, Inc., Redlands, California, USA).

The Luan River located in Hebei Province, China (N41°47′–42°06′, E116°51′–117°45′) was selected as the study area (Fig. 1). The altitude of the study area is from 750 to 1,829 m, and its annual temperature is approximately −1.44 °C, with a maximum temperature of 38.9 °C and a minimum temperature of −42.9 °C. The mean annual precipitation is 454.7 mm, 70% of which occurs from June to August. The mountainous plateau region has a continental monsoon climate and contains seven different soil types (sand soil, meadow soil, boggy soil, black soil, brown soil, cinnamon soil, and grey wooded soil). The characteristic area is a transitional community of warm temperate broad-leaved deciduous forests and temperate grasslands on the eastern plateau.

### Experimental design and data collection

**Figure 2:**
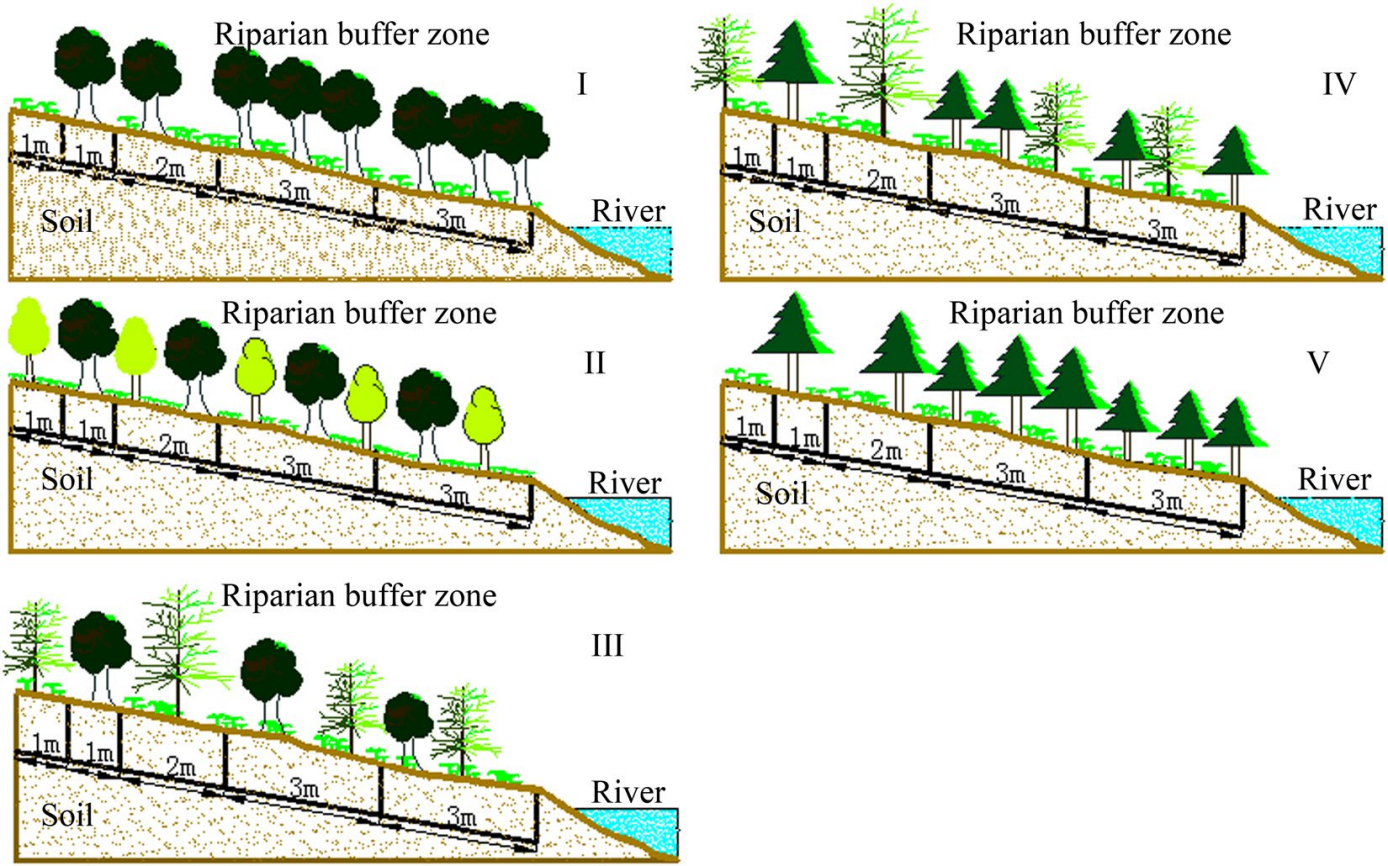
Experimental design and water samples collection. The sketch map of experimental design and water samples collection were generated with AutoCAD 2006 (Autodesk, Inc., San Rafael, California, USA).

Five riparian vegetation configuration patterns were selected to reveal the impacts of riparian vegetation patterns on overland runoff nutrients in the Luan River: I, broad-leaved pure forest dominated by *Betula pendula Roth*.; II, broad-leaved mixed forest composed of *Betula pendula Roth*. and *Betula dahurica Pall*; III, coniferous and broad-leaved mixed forest composed of *Betula pendula Roth*. and *Larix principis-rupprechtii Mayr*.; IV, coniferous mixed forest composed of *Larix principis-rupprechtii* and *Pinus tabulaeformis Carr*.; V, pure coniferous forest dominated by *Larix principis-rupprechtii*. The length and width of transects of the riparian vegetation buffer zone was 10 m (from the upper parts of the riparian vegetation buffer zone to the river channel) (Fig. 2).

The experiment was conducted during the growing season of vegetation (from April to October), and avoided the dormant period of vegetation (from November to March of the following year). This study involved a forestland investigation between April and May in 2016, including topographic features, stand structure characteristics and soil characteristics. The stand structure characteristics of these five vegetation types are shown in Table 2, and their soil characteristics and topographic features are shown in Table 3.

**Table 2:** Characteristics of stands under five vegetation patterns.

Land use types	Diameter at breast height (cm)	Height of tree (m)	Forest density (plants/ha^2^)	Age of stands (a)	Leaf area index	Simpson index (*H´*)	Pielou Index (*Jsi*)
I	12.7 ± 0.31	10.0 ± 0.23	1400	25	1.98 ± 0.01	0.82	0.87
II	13.9 ± 0.15	10.1 ± 0.31	2400	23	2.74 ± 0.08	0.84	0.92
III	18.5 ± 0.21	13.3 ± 0.12	2750	25	3.24 ± 0.03	0.90	0.96
IV	12.7 ± 0.32	11.8 ± 0.21	2200	21	1.78 ± 0.11	0.58	0.82
V	11.8 ± 0.16	11.7 ± 0.33	1200	23	1.61 ± 0.09	0.82	0.61

Note: The data in the table are mean ± SE.

**Table 3:** Characteristics of soil under five vegetation patterns.

Land use types	Altitude (m)	Slope (°)	Soil bulk density (g∙cm^−1^)	< 0.01 mm Physical clay (%)	Soil TN (g∙kg^−1^)	Soil AP (mg∙kg^−1^)	Soil organic matter (g∙kg^−1^)	Soil pH
I	1298	21.0	1.33 ± 0.03	38.58 ± 3.96	1.08 ± 0.29	58.89 ± 4.39	1.05 ± 0.42	6.08 ± 0.06
II	1350	20.5	1.40 ± 0.14	42.55 ± 6.09	1.05 ± 0.52	56.67 ± 3.71	1.08 ± 0.52	6.57 ± 0.09
III	1253	22.0	1.22 ± 0.13	65.51 ± 6.13	1.67 ± 0.42	61.44 ± 1.60	1.67 ± 0.17	6.71 ± 0.08
IV	1281	23.0	1.23 ± 0.11	56.03 ± 6.11	0.64 ± 0.16	51.11 ± 3.06	0.64 ± 0.16	6.71 ± 0.08
V	1240	19.5	1.09 ± 0.04	58.18 ± 18.11	0.65 ± 0.17	70.33 ± 7.58	0.65 ± 0.29	6.47 ± 0.03

Note: The data in the table are mean ± SE.

This study established a small ditch along the four sides of the riparian vegetation buffer zone to separate the study site from the adjacent area. The depth of the ditch was 5 cm, which is the maximum depth of the interaction between runoff and soil. The water discharge experiment was performed with a water aspirator that pumped water from the channel to the upper parts of the riparian vegetation buffer zone at a speed of 10 to 15 m^3^·h^−1^ in the five study areas from June to October 2016. Therefore, the contents of N, P and COD from the river were regarded as the initial concentration values of experiment. To avoid contamination of river water by surface runoff from the buffer zone after purification, water samples were taken from the river channel before each experiment, and the water quality index concentration detected by these water samples was regarded as the initial concentration of surface runoff.

Each experiment started at approximately 10:00 a.m., and each water discharge experiment lasted for 1 h. The water samples were collected from the surface runoff at different widths of the buffer zone with a new ethylene sample bottle (1000 ml per sample) from the upper part of the riparian vegetation buffer zone to the river channel. The widths of the buffer zone are 1 m, 2 m, 4 m, 7 m and 10 m. From June to October 2016, water discharge experiments were carried out in each riparian buffer zone a total of 5 times; a total of 30 water samples were collected from 6 sampling points each time. Therefore, 25 experiments were conducted in total during the experiment period, and a total of 150 water samples were collected for water quality testing.

Before water quality test and analysis, all water samples were filtered through qualitative filter paper, and the filtrate was taken for water quality testing. Before the water samples were tested, they were filtered through qualitative filter paper until the solution was no longer cloudy, and the water samples were colourless. Each index was measured 3 times, and the average value was calculated to represent the water quality index at the sampling point. In this research, COD, TN, TP, AN, and AP were detected in the water samples with an automatic chemistry analyser (Smart Chem 200, Alliance, France).

The data for the concentrations of COD, TN, AN, TP and AP at 0 m, 1 m, 2 m, 4 m, 7 m and 10 m were used to estimate the removal ability of the five riparian vegetation configuration patterns. The removal rate was calculated using the following equation (1):

1 $${E}_{N}=\frac{{C}_{n}-{C}_{0}}{{C}_{n}}\times 100 \% $$

where *E*_*N*_ is the water nutrient indicator (COD, TN, AN, TP and AP) removal rate (%) and *C*_*n*_ is concentration of the water nutrient indicator (COD, TN, AN, TP and AP) (mg·L^−1^) at different widths of the riparian vegetation buffer zone (in this research, n = 0, 1, 2, 4, 7, 10). *C*_0_ is the initial concentration of water nutrient indicator (COD, TN, AN, TP and AP) (mg·L^−1^) replaced by the concentration of river water.

The comprehensive coordinate evaluation method was employed to assess the purification capacity of the five types of riparian buffer zones on surface runoff water quality at different widths, and the evaluation factors included TN (X1), TP (X2), AN (X3), AP (X4) and COD (X5).

First, dimensionless processing was carried out for all data, and the original data table was represented as X_ij_, where i indicated the type of buffer riparian zone and j indicated TN, TP, AN, AP or COD. Second, each data point was compared to the maximum value (X_j_) for that index to construct the matrix coordinate as represented by d_ij_ using the following equation (2):

2 $${d}_{ij}=\frac{{X}_{ij}}{{X}_{j}}$$

The distance of the i index from the standard point was then calculated using the following equation (3), and the next step was to calculate the sum of the distances of each treatment to the standard point using the following equation (4). Finally, the order was ranked according to the M value, and the smallest value among the comprehensive results was considered best.

3 $${p}_{i}=\sqrt{\sum _{i}\,{(1-{d}_{ij})}^{2}}$$

4 $$M=\sum _{i=1}^{n}\,{p}_{i}$$

where n is the numbers of indicators evaluated in this study, n = 5.

I, broad-leaved pure forest dominated by *B. pendula*; II, broad-leaved mixed forest composed of *B. pendula* and *B. dahurica*; III, coniferous and broad-leaved mixed forest composed of *B. pendula* and *L. principis-rupprechtii*; IV, coniferous mixed forest composed of *L. principis-rupprechtii* and *P. tabulaeformis*; V, pure coniferous forest dominated by *L. principis-rupprechtii*.

### Data analysis

Differences in the measured parameters among the different samples were analysed using one-way analysis of variance and Fisher’s protected least significant difference test. All statistical analyses were conducted using SPSS software (v. 19.0, SPSS Inc., Chicago, IL, USA), at the *P* = 0.05 level of significance. The sketch map of experimental design and water samples collection were generated with AutoCAD 2006 (Autodesk, Inc., San Rafael, California, USA). The comprehensive evaluation of water nutrients in runoff was conducted using PCA; the higher comprehensive score indicates a better purification effect on water nutrients of a riparian buffer zone.

## Results

### Distribution characteristic of surface runoff water nutrients

**Figure 3:**
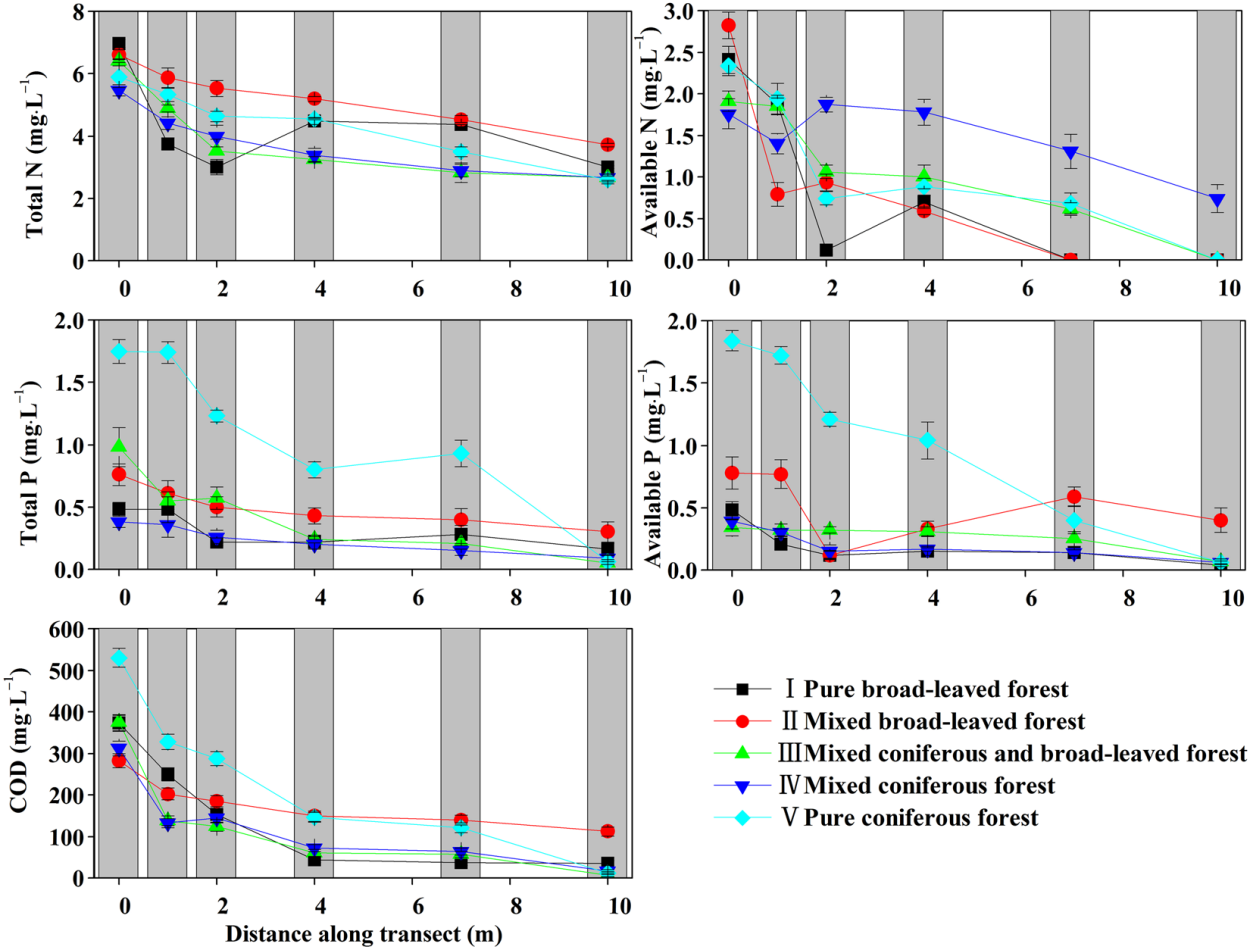
Distribution of concentrations of COD, TN, TP, AP and AN in different width transect of five riparian vegetation buffer zones.

The variability in the concentrations of COD, TN, AN, TP and AP from runoff water under different vegetation patterns are shown in Fig. 3, and the findings indicated a decreasing trend in the five different riparian vegetation buffer zones from the upper parts of the riparian vegetation buffer zone to the river channel. Both the highest and the lowest TN concentration (6.97 ± 0.14 mg·L^−1^ and 3.01 ± 0.05 mg·L^−1^, respectively) were found in the broad-leaved forest dominated by *B. pendula* at 10 m and 0 m from the upper part of the riparian vegetation buffer zone to the river channel. Similarly, the highest and lowest AN concentration (2.82 ± 0.16 mg·L^−1^ and 0.00 ± 0.00 mg·L^−1^, respectively) were detected in the mixed broad-leaved forest composed of *B. pendula* and *B. dahurica* at 10 m, 3 m and 0 m from the upper part of the riparian vegetation buffer zone to the river channel. The highest TP and AP concentrations (1.75 ± 0.09 mg·L^−1^ and 1.84 ± 0.08 mg·L^−1^ at 10 m from the upper part of the riparian vegetation buffer zone to the river channel) and the lowest TP and AP concentrations (0.06 ± 0.02 mg·L^−1^ and 0.04 ± 0.01 mg·L^−1^, respectively, at 0 m from the river) were detected in the pure coniferous forest (dominated by *L. principis-rupprechtii*). Figure 3 also illustrates the highest COD concentration (530 ± 50.52 mg·L^−1^) and the lowest COD concentration (12.4 ± 1.6 mg·L^−1^), which were measured in the pure coniferous forest dominated by *L. principis-rupprechtii* at 10 m and at 0 m from the upper part of the riparian vegetation buffer zone to the river channel.

### Comparison of the average nutrient contents of overland runoff

**Figure 4:**
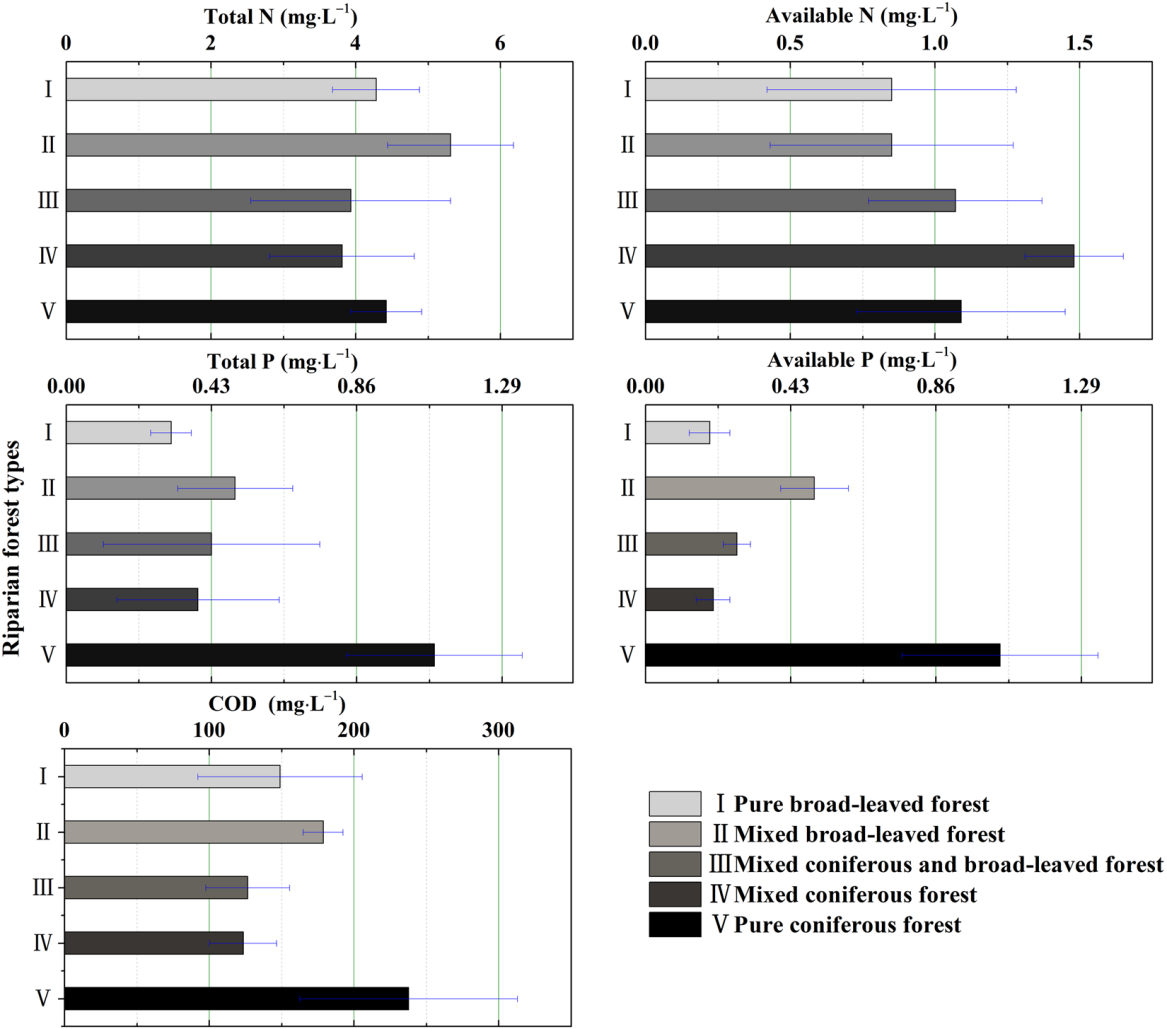
Compare the mean value of concentrations of COD, TN, TP, AP and AN in five riparian vegetation configuration patterns.

The concentrations of COD, TN, AN, TP and AP from runoff water differed among the riparian vegetation configuration patterns (Fig. 4). The highest mean TN (5.31 ± 0.87 mg·L^−1^) appeared in riparian vegetation pattern II (mixed broad-leaved forest composed of *B. pendula* and *B. dahurica*), and the lowest mean TN (3.81 ± 0.09 mg·L^−1^) appeared in riparian vegetation pattern IV (mixed coniferous forest composed of *L. principis-rupprechtii* and *P. tabulaeformis*) (^*^P > 0.05). However, the highest mean AN (1.48 ± 0.17 mg·L^−1^) appeared in riparian vegetation pattern IV (mixed coniferous forest composed of *L. principis-rupprechtii* and *P. tabulaeformis*), and the lowest mean AN (0.85 ± 0.42 mg·L^−1^) appeared in riparian vegetation pattern II (mixed broad-leaved forest composed of *B. pendula* and *B. dahurica*). The highest mean TP and mean AP (1.09 ± 0.26 mg·L^−1^ and 1.05 ± 0.29 mg·L^−1^, respectively) appeared in riparian vegetation pattern V (pure coniferous forest dominated by *L. principis-rupprechtii*), and the lowest mean TP and mean AP (0.31 ± 0.06 mg·L^−1^ and 0.19 ± 0.06 mg·L^−1^, respectively) appeared in riparian vegetation pattern I (pure broad-leaved forest dominated by *B. pendula*) (^*^P > 0.05). The highest mean COD (237.57 ± 75.16 mg·L^−1^) appeared in riparian vegetation pattern V (pure coniferous forest dominated by *L. principis-rupprechtii*), and the lowest mean COD (123.45 ± 23.05 mg·L^−1^) appeared in riparian vegetation pattern IV (mixed coniferous forest composed of *L. principis-rupprechtii* and *P. tabulaeformis*) (^*^P > 0.05).

### Purification ability of a single parameter

**Figure 5:**
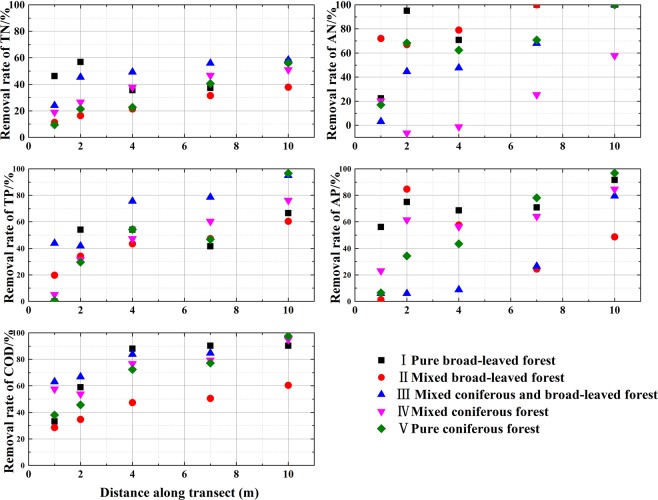
Removal rates of COD, TN, AN, TP and AP in different riparian vegetation patterns.

The removal rates of COD, TN, AN, TP and AP in the five riparian vegetation configuration patterns are shown in Fig. 5. The ability to remove COD, TN, AN, TP and AP from runoff water gradually increased from the upper part of the riparian vegetation buffer zone to the river channel. When the removal rates of the nutrient indicators (TN, AN, TP, AP and COD) in the five riparian vegetation configuration patterns were compared, the significant removal effectiveness of TN and AN as well as COD appeared in riparian vegetation pattern III, with removal rates of 58.41%, 100%, 98.27%, respectively. Accordingly, TN, AN, COD were adsorbed at an average rate of 0.41 (mg∙L^−1^∙m^−1^), 0.30 (mg∙L^−1^∙m^−1^) and 56.65 (mg∙L^−1^∙m^−1^), respectively, along the transect. The highest removal effectiveness of TP and AP appeared in riparian vegetation pattern V, with removal rates of 58.41% and 100%, respectively. Correspondingly, TP and AP were adsorbed at an average rate of 0.18 (mg∙L^−1^∙m^−1^) and 0.19 (mg∙L^−1^∙m^−1^), respectively, along the transect.

### Comprehensive purification ability of five riparian vegetation buffer zones

This study measured the parameters COD, TN, AN, TP and AP as indicators to evaluate the runoff purification abilities of different riparian vegetation buffer zones at 1 m, 2 m, 4 m, 7 m and 10 m by applying the comprehensive coordinate method. The comprehensive evaluation results indicated that the runoff COD, TN, AN, TP and AP purification capacities at 1 m, 2 m, 4 m, 7 m and 10 m differed among the five buffer riparian zones (Table 4). Ranking in terms of the removal of COD, TN, AN, TP and AP from runoff water at 1 m and 2 m showed that riparian vegetation pattern I showed the highest purification ability; patterns II and IV had the lowest impact.

**Table 4:** Comprehensive purification effect ranking of five riparian vegetation buffer zones at different widths.

Width (m)	Forest pattern	TN	TP	AP	AN	COD	Comprehensive evaluation result	Purification effect rank
1	I	0.00	0.00	0.00	0.01	0.00	**0.01**	1
	II	0.36	0.68	0.08	0.06	0.63	1.81	5
	III	0.06	0.77	0.00	0.13	0.52	1.48	3
	IV	0.15	0.31	0.35	0.00	0.42	1.23	2
	V	0.34	0.66	0.00	0.03	0.47	1.50	4
2	I	0.00	0.00	0.37	0.03	0.00	**0.39**	1
	II	0.13	0.81	0.25	0.00	0.35	1.54	3
	III	0.32	0.54	0.19	0.13	0.29	1.47	2
	IV	0.22	0.81	0.88	0.21	0.67	2.79	5
	V	0.25	0.77	0.00	0.08	0.62	1.72	4
4	I	0.00	0.47	0.45	0.00	0.21	1.13	2
	II	0.49	0.73	0.37	0.02	0.53	2.14	5
	III	0.00	0.00	0.26	0.01	0.00	**0.27**	1
	IV	0.26	0.70	0.00	0.12	0.56	1.65	3
	V	0.35	0.49	0.19	0.14	0.49	1.67	4
7	I	0.29	0.58	0.00	0.13	0.70	1.71	3
	II	0.55	0.58	1.00	0.00	0.49	2.62	5
	III	0.00	0.00	1.00	0.00	0.32	1.32	2
	IV	0.35	0.33	0.29	0.14	0.60	1.71	4
	V	0.02	0.10	0.23	0.05	0.00	**0.40**	1
10	I	0.79	0.72	1.00	0.09	0.64	3.24	5
	II	0.89	0.68	1.00	0.08	0.69	3.34	4
	III	0.73	0.72	0.00	0.08	0.49	2.03	2
	IV	0.00	0.00	1.00	0.00	0.00	**1.00**	1
	V	0.46	0.81	1.00	0.04	0.22	2.52	3

However, at a width of 4 m, riparian vegetation pattern III had the highest purification ability, and pattern II still had the lowest impact. At a distance of 7 m, riparian vegetation pattern V exhibited the highest purification ability and pattern II the lowest. At 10 m, riparian vegetation pattern IV showed the highest purification ability and pattern II the lowest.

## Discussion

### Effects of riparian buffer vegetation zone width on nutrient removal from runoff water

The purification of runoff water revealed a positive relationship with the riparian vegetation buffer zone width^20–22^. Identifying the proper width of the riparian vegetation buffer zone is a key strategy to improving nutrient removal ability^23,24^. The concentrations of COD, TN, AN, TP and AP in the five riparian vegetation configuration patterns gradually declined from the upper parts of the vegetation zone to the river channel. In addition, this research showed that the concentrations of COD, TN, AN, TP and AP at different sites (0 m, 1 m, 2 m, 4 m, 7 m, 10 m) were very different in the five riparian vegetation configuration patterns (Fig. 3). Logically, the element concentration should be reduced after purification by the buffer zone, while the concentration of elements along the transect increases locally, which is attributed to the contamination of groundwater^25,26^. Differences among the purification effectiveness may be attributed to mechanisms in each forest buffer. Settling, infiltration, and dilution processes can explain this change trend. When surface water flows through the buffer zone along the river bank, it moves vertically along the soil pore in addition to the horizontal movement, and the two movements always coexist in the case of water flow. The water movement in these two directions has an effect on leaching^26^, settling^27,28^, infiltration^29^, and dilution^9,25^. The elements originally contained in soil are washed and transported to the next location under the action of water dissolution and force^30–33^, which is also the reason for the increased element concentration in certain water sampling points in Fig. 3. Overall, the concentrations of these elements were decreased after the purification of the buffer zone in this study, and the purification effectiveness reached 30–80%.

Generally, the distance from the channel to the riparian vegetation buffer zone bank is not great. Therefore, the risk of river pollution from soil and water erosion is higher in these areas, and the management of these five riparian forests should be considered to strengthen management. Previous studies have found a decline in nutrients with distance from the river^34,35^. Additionally, there was a close relationship between nutrient removal capacity and the absorption or transformation of potential pollutants^36^. Therefore, the width variation and vegetation characteristics are important factors in understanding riparian vegetation buffer zone purification ability.

### Effects of riparian buffer zone vegetation and soil on nutrient removal from runoff water

Identifying rational vegetation configuration patterns to control river pollution is imperative^37^. Previous studies have found that vegetation configuration patterns have a remarkable effect on the distribution and removal capacity of TN and TP^15,18,38–41^. The comprehensive evaluation results of this study showed that different plants show different purification ability with respect to TN, AN, TP, AP and COD. The results (Table 4), indicate a high removal effectiveness under vegetation type I (broad-leaved pure forest dominated by *B. pendula*) at a distance of 0 to 2 m. However, from 2 m to 4 m, vegetation type III showed the maximum removal effectiveness (coniferous and broad-leaved mixed forest composed of *B. pendula*), while at a distance of 4 m to 7 m, the highest removal rate was achieved in vegetation type V (pure coniferous forest dominated by *L. principis-rupprechtii*). From 7 m to 10 m, vegetation type IV presented the maximum removal effectiveness (coniferous mixed forest composed of *L. principis-rupprechtii* and *P. tabulaeformis*).

Obviously, the removal effectiveness is very different in the five types of forested riparian zones in this study. The nitrogen and phosphorus leaching from riparian zones depended significantly on vegetation type^26,42^. There is a complex relationship between vegetation and soils. Vegetation composition not only affects the soil properties but also affects the microclimate in the forest. In this research, the stand characteristics of these five vegetation patterns differed widely (Table 2). The leaf area index (3.24 ± 0.03) and forest density (2750 plants/hm^2^) of the forested riparian zone III was greater than in the other four forested riparian zones, which is beneficial to increasing the amount of litter and organic matter because of the leaves and wood deposited on and decomposed in the soils. The organic matter (1.67±0.17 g⋅kg^−1^) of the forested riparian zone III was greater than in the other four forested riparian zones (Table 3). A higher content of organic matter could reinforce nitrogen and phosphorus of adsorption and retention from surface runoff water. The removal effectiveness of the forested riparian zone III validates this view (Table 4). The results of this study corroborate previous work showing that the forested riparian zones can retain water and contaminants associated with agricultural runoff^38^. In addition, the Simpson index (0.90) and Pielou Index (0.96) of the forested riparian zone III were higher than in the other four forested riparian zones, indicating abundant herbs and fine roots (root diameter ≤3 mm). Plant roots are the main carrier of microbial species^27,34^, and microorganism growth on the surface of these roots is greater because of the larger effective space and opportunity for attachment of microorganisms and nutrient uptake. This phenomenon is beneficial to the processes of nitrification/denitrification, which can effectively promote the removal effectiveness of water purification^26,27,29^.

A previous study showed that certain biological processes can explain the purification mechanism involved in the removal of nitrogen and phosphorous in soil, such as infiltration, gravitational settlement, interception, ammonification, nitrification, denitrification, and uptake by vegetation^27^. Therefore, the soil provides important microenvironments supporting those biological processes^11,27^. Furthermore, the removal of nitrogen and phosphorus is often closely bound to soil particles^29^. Compared with that in the other four forested riparian zones, the physical clay (<0.01 mm) of the forested riparian zone III was largest (65.51 ± 6.13), the soil bulk density of the forested riparian zone III was in the middle of the five zones (1.22 ± 0.13), and the organic matter (1.67 ± 0.17 g⋅kg^−1^) of the forested riparian zone III was highest, which indicates that the soil conditions of the forested riparian zone III are beneficial for infiltration, gravitational settlement, interception, ammonification, nitrification, denitrification, and uptake by vegetation. Phosphorus leaching loss from riparian zones depended significantly on vegetation types^42^. The average removal rate of nitrogen and phosphorus as well as chemical oxygen demand are equal to the ratio (*V* = *ΔC*/*D*) of the difference (*ΔC* = *C*_10_ − *C*_0_) between the concentration at the entrance and at the exit with the width (*D* = 10 m) of the riparian vegetation buffer zone. Thus, the pure coniferous forest can absorb P at a rate of 0.19 (mg∙L^−1^∙m^−1^) along the transect, as illustrated in Figs 3 and 5. The results of this study corroborate previous work showing that the forested riparian zones can retain water and contaminants associated with agricultural runoff^27,43,44^.

Water purification is strongly related to the functional characteristics of the plants. In present study, the results demonstrated a vegetation configuration pattern with a highly efficient purification capacity based on comprehensive evaluation results regarding the removal of nitrogen and phosphorous and chemical oxygen demand. However, how to select plants that can increase the nutrient removal rate in forested buffer zones has not been determined. According to the present results, plant species with larger fine-root biomass should be considered first in the selection of vegetation for the restoration or construction of forest riparian buffer zones.

## Conclusion

In this study, runoff water quality measurements of TN, AN, TP, AP and COD were recorded in five riparian vegetation configuration patterns in the Luan River in China. The aim was to identify a pattern with a high-efficiency purification ability for these pollutants. The results showed that vegetation configuration types may have a crucial effect on COD, TN, AN, TP and AP. There was a decreasing trend for all concentrations from the upper part of the riparian buffer zone to the river channel, and the different forest types showed varying abilities to remove these nutrients.

In terms of the removal rate of a single indicator, vegetation pattern III exhibited the highest purification ability for TN and COD (with removal rates of 58.41% and 98.27%, respectively) among the studied vegetation patterns. In addition, vegetation pattern V had a higher purification ability for TP and AP (with removal rates of as high as 96.56% and 96.74%, respectively) than did other vegetation patterns. Additionally, vegetation patterns I, II and V presented higher purification abilities for AN (the removal rate was as high as 100%).

From the upper part of the riparian buffer zone to the river channel, the comprehensive evaluation results showed that pattern I had the greatestest purification ability at 1 m and 2 m, at a width of 4 m, pattern III had the highest purification ability. At a distance of 7 m, riparian vegetation pattern V showed the highest purification ability, however, pattern IV showed the highest purification ability and pattern II the lowest at 10 m. It is recommended that planting broad-leaved species (such as *B. pendula* and *B. dahurica*) be prioritized from 0 m to 4 m (from the upper part of the riparian buffer zone to the river channel) and that conifer species (such as *L. principis-rupprechtii* and *P. tabulaeformis*) be prioritized from 4 m to 10 m. Constructing a complex stand configuration and stand structure will improve the ecological function of the riparian buffer zone and thus surface runoff purification capacity. Therefore, this study suggests the use of these vegetation configuration patterns in the development of runoff purification systems.
